# Msb1 Interacts with Cdc42, Boi1, and Boi2 and May Coordinate Cdc42 and Rho1 Functions during Early Stage of Bud Development in Budding Yeast

**DOI:** 10.1371/journal.pone.0066321

**Published:** 2013-06-13

**Authors:** Yuan Liao, Fei He, Ting Gong, Erfei Bi, Xiang-Dong Gao

**Affiliations:** 1 State Key Laboratory of Virology, College of Life Sciences, Wuhan University, Wuhan, China; 2 Department of Cell and Developmental Biology, University of Pennsylvania Perelman School of Medicine, Philadelphia, Pennsylvania, United States of America; Texas A&M University, United States of America

## Abstract

Msb1 is not essential for growth in the budding yeast *Saccharomyces cerevisiae* since *msb1*Δ cells do not display obvious phenotypes. Genetic studies suggest that Msb1 positively regulates Cdc42 function during bud development, since high-copy *MSB1* suppressed the growth defect of temperature-sensitive *cdc24* and *cdc42* mutants at restrictive temperature, while deletion of *MSB1* showed synthetic lethality with *cdc24*, *bem1*, and *bem2* mutations. However, the mechanism of how Msb1 regulates Cdc42 function remains poorly understood. Here, we show that Msb1 localizes to sites of polarized growth during bud development and interacts with Cdc42 in the cells. In addition, Msb1 interacts with Boi1 and Boi2, two scaffold proteins that also interact with Cdc42 and Bem1. These findings suggest that Msb1 may positively regulate Cdc42 function by interacting with Cdc42, Boi1, and Boi2, which may promote the efficient assembly of Cdc42, Cdc24, and other proteins into a functional complex. We also show that Msb1 interacts with Rho1 in the cells and Msb1 overproduction inhibits the growth of *rho1-104* and *rho1-3* but not *rho1-2* cells. The growth inhibition appears to result from the down-regulation of Rho1 function in glucan synthesis, specifically during early stage of bud development. These results suggest that Msb1 may coordinate Cdc42 and Rho1 functions during early stage of bud development by promoting Cdc42 function and inhibiting Rho1 function. Msb1 overproduction also affects cell morphology, septin organization, and causes increased, aberrant deposition of 1,3-β-glucan and chitin at the mother-bud neck. However, the stimulation of glucan synthesis mainly occurs during late, but not early, stage of bud development.

## Introduction

Rho GTPases in eukaryotic cells are key regulators of cytoskeletal rearrangement and membrane trafficking. In the budding yeast *Saccharomyces cerevisiae*, the Rho GTPase Cdc42 and Rho1 are essential for bud development [1]. Cdc42 is thought to be specifically required for the initiation of bud assembly, the first step of bud development, because temperature-sensitive (Ts^−^) *cdc42* mutant, such as *cdc42-1*, could not restrict cell surface growth to the budding site at restrictive temperature. The cells grow isotropically and become large and round without a bud while the nuclear cell cycle continues [2]. Cdc42 is known to control polarized organization of the actin cytoskeleton and secretion [2], [3]. In addition, Cdc42 controls the assembly of the septin cytoskeleton at the mother-bud neck [4], which serves as a diffusion barrier preventing the diffusion of molecules from the daughter cell surface into the mother cell [5].

Cdc42 localizes to the plasma membrane and endomembranes and clusters at sites of polarized growth, including the incipient bud site before bud emergence, the bud tip and side of small- and medium-budded cells, and the mother-bud neck in large-budded cells [6]. Like other Rho GTPases, Cdc42 is positively regulated by the guanine nucleotide exchange factor (GEF), Cdc24, which promotes the binding of Cdc42 to GTP. The four GTPase-activating proteins (GAPs), Rga1, Rga2, Bem2, and Bem3, negatively regulate Cdc42 by increasing the intrinsic GTPase activity of Cdc42 [1]. Genetic studies also identified Msb1 as a positive regulator of Cdc42 function. *MSB1* was first identified as a high-copy suppressor of the temperature-sensitive growth defect of *cdc24-4* mutant, which was also suppressed well by high-copy *CDC42* [7]. High-copy *MSB1* also suppressed several *cdc42*-Ts mutants, including *cdc42-1* [7], [8], *cdc42-201* [9], and *cdc42^G60D^* [10], as well as Ts^−^ *bem4*Δ [11] and *gic1 gic2* mutants [12]. Bem4, Gic1, and Gic2 all physically interact with Cdc42. Because all these mutants are defective in cell polarity establishment and bud emergence, these data suggest that Msb1 plays a significant role in the initiation of bud assembly. Gene deletion studies indicate that *MSB1* is dispensable for cell growth or bud formation under normal condition but becomes essential for growth in cells bearing temperature-sensitive *cdc24*, *bem1* or *bem2* mutation [13]. *BEM1* encodes a scaffold protein critical for Cdc42 activation whereas *BEM2* encodes a RhoGAP for Cdc42, Rho1, and Rho4 [1], [14]. Both *BEM1* and *BEM2* are involved in bud formation. This finding further supports that Msb1 positively regulates Cdc42 function. However, the mechanism is not known.

Like Cdc42, Rho1 also plays a role in actin organization and secretion since certain *rho1*-Ts mutants, such as *rho1-2*, displayed an actin-organization defect and depolarized growth at restrictive temperature [15]. However, Rho1’s major function appears to be in the control of 1,3-β-glucan synthesis in the cell wall because several *rho1*-Ts mutants, such as *rho1-3* and *rho1-104*, often died of cell lysis at the small bud stage at restrictive temperature [15]–[18]. *MSB1* also genetically interacts with genes involved in Rho1-mediated cell wall synthesis. The yeast 1,3-β-glucan synthase is made of one catalytic subunit, Fks1/Fks2, and one regulatory subunit, Rho1 [17], [18]. *MSB1* was identified as a high-copy suppressor of temperature-sensitive growth defect of *fks1-1154 fks2*Δ mutant at 34°C, a mutant defective in the synthesis of 1,3-β-glucan in the cell wall [19]. This finding suggests that Msb1 may act positively on 1,3-β-glucan synthase. The same study also showed that high-copy *MSB1* suppressed the growth defect of *rho1-2* mutant at 37°C. However, the mechanism of this genetic interaction with *RHO1* is not clear.

Here, we show that Msb1 localizes to sites of polarized growth and interacts with Cdc42 in the cells. Msb1 also interacts with Boi1 and Boi2, two Cdc42-interacting proteins. Thus, Msb1 may promote Cdc42 function by interacting with Cdc42, Boi1, and Boi2. In addition, we show that Msb1 interacts with Rho1 in the cells and Msb1 overproduction inhibits Rho1 function in glucan synthesis in small-budded cells. Our findings suggest that Msb1 may play a role in the coordination of Cdc42 and Rho1 functions during early stage of bud development.

## Results

### Msb1 Localizes to Sites of Polarized Growth and Interacts with Cdc42 *in vivo*

**Figure 1:**
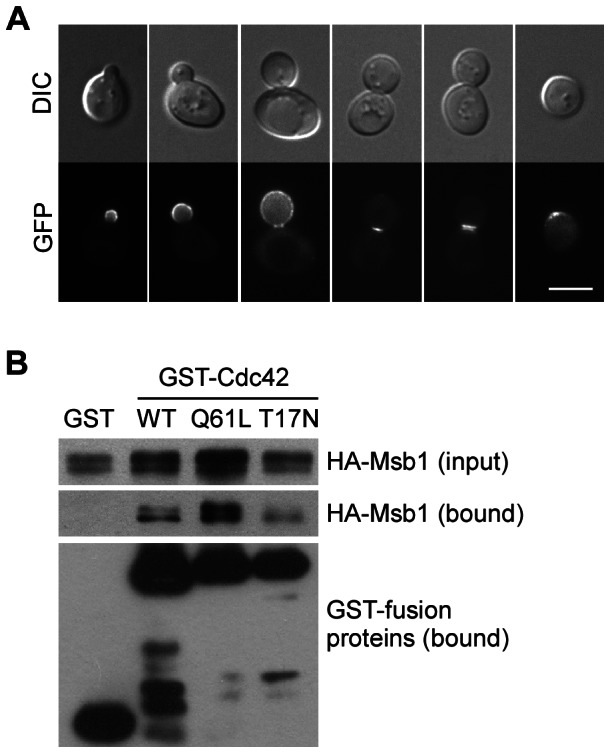
Msb1 localizes to sites of polarized growth and interacts with Cdc42. (**A**) GFP-Msb1 localization during bud development. Cells of yeast strain YEF1395 (*msb1*Δ) carrying plasmid pRS426-GFP-MSB1 were grown in SC-Ura medium and examined for GFP fluorescence. Bar, 5 µm. (**B**) Msb1 interacts with Cdc42 by GST pull-down assay. Cells of yeast strain YEF473A carrying YEp181-3HA-MSB1 along with pEGKT (GST), pEGKT-CDC42 (GST-Cdc42, WT), pEGKT-CDC42^Q61L^ (GST-Cdc42, Q61L), or pEGKT-CDC42^T17N^ (GST-Cdc42, T17N) were grown in SC-Leu-Ura medium containing 2% raffinose at 30°C. Galactose was added to a final concentration of 2%, and the cultures were grown for 4 h to induce the expression of GST-fusion proteins. GST or GST-tagged proteins were pulled down by glutathione-Sepharose beads from equal amounts of Triton X-100-solubilized cell lysates. Molecular weight: GST (27 kDa), GST-Cdc42 (46 kDa), HA-Msb1 (130 kDa).

To gain some clues on how Msb1 regulates Cdc42 function, we first examined the localization of Msb1 in yeast cells. Since Msb1 is a protein of low abundance [20], we expressed an N-terminally tagged *GFP-MSB1* construct under the control of its endogenous promoter on a high-copy plasmid. This construct was functional because it could suppress the *cdc42-201* mutant (data not shown). We observed that GFP-Msb1 localized to sites of polarized growth on the cell surface in a cell cycle-dependent manner (Fig. 1A): Msb1 localized to a patch at the presumptive bud site. After bud emergence, Msb1 localized to the entire bud cortex in the small bud. As the bud enlarged to a medium size, Msb1 gradually disappeared from the bud cortex and relocated to the mother-bud neck. During cytokinesis, Msb1 at the bud neck split into two rings. After cell separation, the daughter cell and the mother cell each inherited one ring or patch, which persisted for a short period of time.

The localization pattern of Msb1 is similar to that of Cdc42 [6]. This prompted us to investigate if Msb1 might associate with Cdc42 in yeast cells. To this end, we expressed Msb1 tagged with three copies of HA at its N-terminus in the cells. Cdc42 was tagged with GST. Glutathione-Sepharose beads were used to pull down GST-Cdc42 and an anti-HA antibody was used to detect the presence of HA-Msb1 in the precipitates. Our result showed that Msb1 could be pulled down with Cdc42, but not with GST alone (Fig. 1B), indicating that Msb1 interacts with Cdc42 *in vivo*. In addition, Msb1 appeared to be pulled down more efficiently with Cdc42^Q61L^, a constitutively active mutant that mimics the GTP-bound form of Cdc42, than with Cdc42^T17N^, a mutant that mimics the GDP-bound form. Taken together, our findings reveal that Msb1 localizes to sites of polarized growth and interacts with Cdc42 *in vivo*.

### Interaction of Msb1 with Proteins that Bind Cdc42

**Figure 2:**
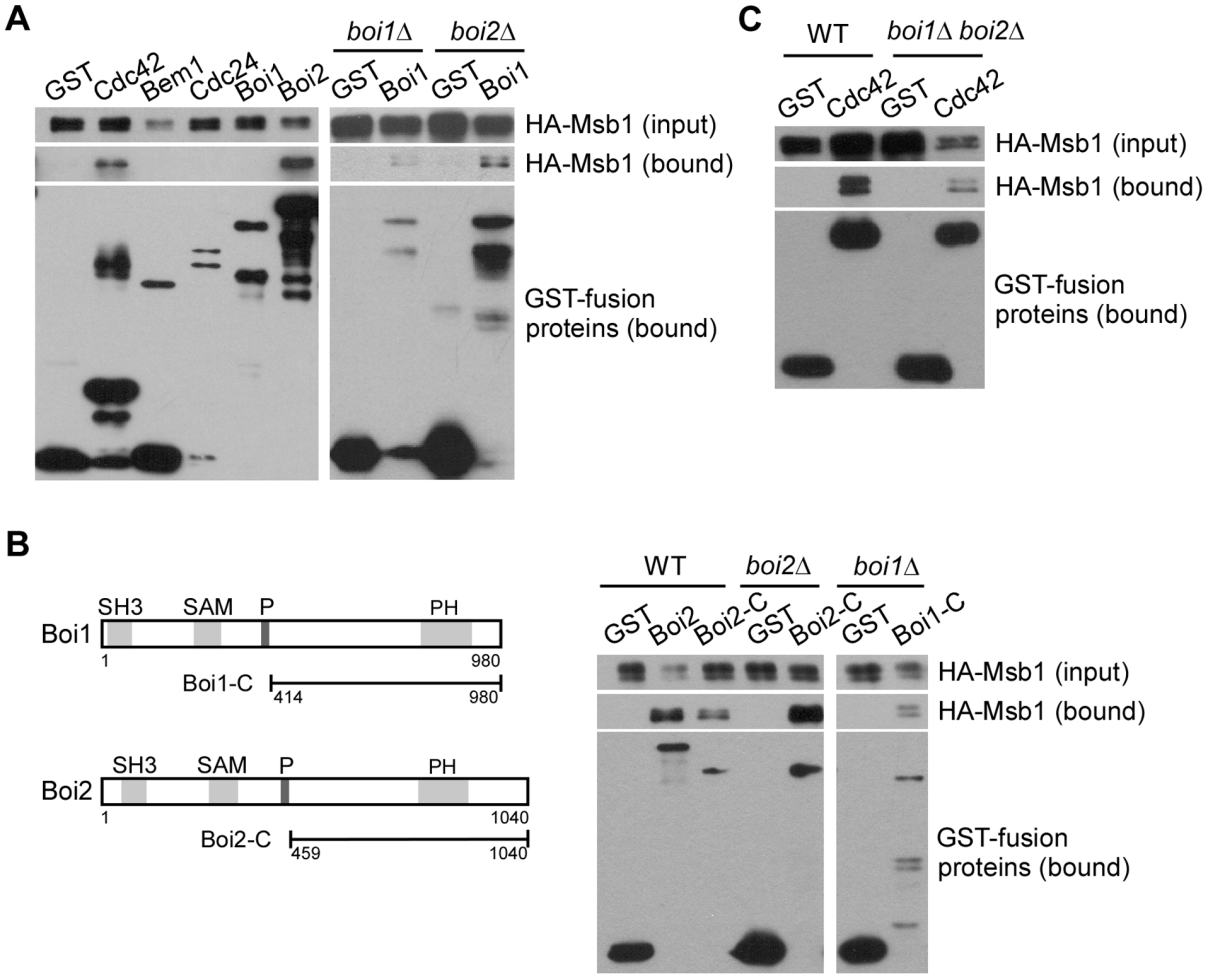
Detection of Msb1 interaction with Bem1, Cdc24, Boi1, and Boi2. (**A**) Msb1 interacts with Boi2 and Boi1 by GST pull-down assay. Cells of yeast strain YEF1395 (*msb1*Δ) carrying YEp181-3HA-MSB1 along with pEGKT (GST), pEGKT-CDC42 (Cdc42), pEGKT-BEM1 (Bem1), pEGKT-CDC24 (Cdc24), pEGKT-BOI1 (Boi1), or pEGKT-BOI2 (Boi2), as well as cells of yeast strain JGY2425 (*boi1*Δ) or JGY2349 (*boi2*Δ) carrying YEp181-3HA-MSB1/pEGKT or YEp181-3HA-MSB1/pEGKT-BOI1 were used in the assay. (**B**) Msb1 interacts with the C-terminal region of Boi1 and Boi2 lacking the proline-rich motif. Left panel, the schematic diagram of domain structure in Boi1 and Boi2. P, proline-rich motif. Right panel, GST pull-down assay with GST-tagged Boi2, Boi2-C, and Boi1-C in yeast strains YEF473A (WT), JGY2425 (*boi1*Δ), and JGY2349 (*boi2*Δ). (**C**) Msb1 interacts with Cdc42 in *boi1*Δ *boi2*Δ cells. GST pull-down assay was performed in cells of yeast strain YEF473A (WT) and JGY2821 (*boi1*Δ *boi2*Δ) carrying YEp181-3HA-MSB1/pEGKT or YEp181-3HA-MSB1/pEGKT-CDC42. Molecular weight of GST-tagged proteins: Cdc42 (46 kDa), Bem1 (86 kDa), Cdc24 (120 kDa), Boi1 (133 kDa), Boi2 (140 kDa), Boi1-C (88 kDa), and Boi2-C (90 kDa).

To understand how Msb1 positively regulates Cdc42 function, we also examined if Msb1 interacts with proteins that are known to bind Cdc42, such as Cdc24 and Bem1, whose coding genes also genetically interact with *MSB1*. In addition, we wanted to examine if Msb1 interacts with Boi1 and Boi2, two closely related scaffold proteins that bind Cdc42 and Bem1 and play important roles in the establishment of cell polarity [21], [22]. To this end, we tagged Cdc24, Bem1, Boi1, and Boi2 with GST at their N-terminuses. GST pull-down assay was used to investigate the interaction between Msb1 and these proteins (Fig. 2A, left panel). We found that Msb1 was efficiently pulled down by Boi2, indicating that Msb1 interacts with Boi2 *in vivo*. However, we did not detect an interaction of Msb1 with Cdc24, Bem1, or Boi1.

Boi1 and Boi2 are highly homologous to each other and are thought to play redundant roles in cell polarization [21], [22]. The failure to detect an interaction between Msb1 and Boi1 could be that Boi1-GST may not be efficient to compete with endogenous Boi1 or Boi2 for binding to Msb1. We then repeated the pull-down assay in *boi1*Δ and *boi2*Δ cells. As expected, Msb1 was pulled down by Boi1 in *boi1*Δ and *boi2*Δ cells albeit less efficiently than Msb1 was pulled down by Boi2 (Fig. 2A, right panel), indicating that Msb1 also interacts with Boi1 *in vivo*.

Boi1 and Boi2 are large scaffold proteins with multiple domains that mediate the interaction with other proteins. Each of them carries a SH3 domain, a SAM (sterile alpha motif) domain, a proline-rich motif, and a PH domain (Fig. 2B, left panel). It is known that Boi1 and Boi2 bind to Bem1 via their proline-rich motifs, which bind to the second SH3 domain of Bem1, whereas they interact with Cdc42 via their C-terminal PH domain-bearing region lacking the Bem1-binding site [22]. To determine the region in Boi1 and Boi2 that interacts with Msb1, we performed a pull-down assay using the C-terminal segment of Boi1 and Boi2 that lacks the Bem1-binding site. We found that Msb1 can be pulled down by Boi2-C (aa 459–1040) in wild-type and *boi2*Δ cells (Fig. 2B, right panel). Msb1 can also be pulled down by Boi1-C (aa 414–980) in *boi1*Δ cells. The interaction between Msb1 and Boi1/Boi2 raised a possibility that Boi1 and Boi2 may play a mediator role in the interaction between Msb1 and Cdc42. We found that this is unlikely the case because Msb1 could be pulled down by Cdc42 in *boi1*Δ *boi2*Δ cells (Fig. 2C). Together, these results suggest that Msb1 interacts with Boi1 and Boi2 in yeast cells, and the C-terminal region of Boi1 and Boi2 is sufficient for their interaction with Msb1.

### Functional Interaction between Msb1 and Boi1/Boi2

**Figure 3:**
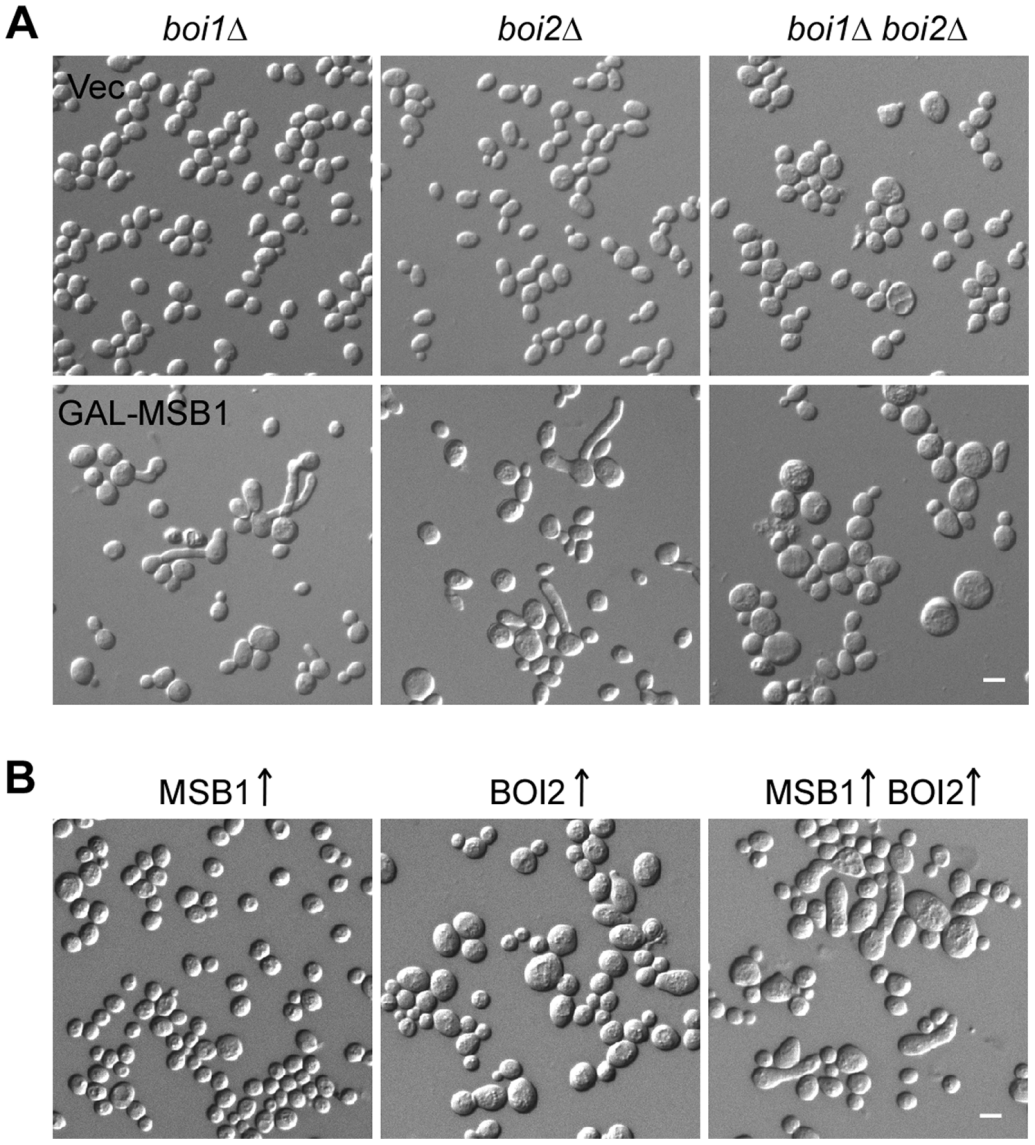
Functional interaction between Msb1 and Boi1/Boi2. (**A**) Morphology of *boi1*Δ, *boi2*Δ, and *boi1*Δ *boi2*Δ cells overexpressing *MSB1*. Cells of yeast strains JGY2425 (*boi1*Δ), JGY2349 (*boi2*Δ), and JGY2821 (*boi1*Δ *boi2*Δ) carrying pEGKT316 (Vec) or pEGKT316-MSB1 (GAL-MSB1) were grown in SC-Ura medium containing galactose and raffinose for 12 h. (**B**) Morphology of cells with elevated expression of *MSB1* and *BOI2*. Cells of yeast strain YEF473A carrying plasmids YEp13-MSB1/pUG36 (MSB1↑), YEp13/pUG36-BOI2 (BOI2↑), or YEp13-MSB1/pUG36-BOI2 (MSB1↑ BOI2↑) were grown on SC-Leu-Ura plate containing dextrose at 30°C for 16 h. Bars, 5 µm.

*GAL1* promoter-driven overexpression of *MSB1* in yeast cells caused bud elongation and the formation of multiple buds (see next section). We found that *GAL1*-driven overexpression of *MSB1* also produced elongated buds in *boi1*Δ and *boi2*Δ cells but not in *boi1*Δ *boi2*Δ cells (Fig. 3A). The percentages of budded cells with an elongated bud are 20% (n = 200) for *boi1*Δ cells, 24% (n = 200) for *boi2*Δ cells, but 0% (n = 200) for *boi1*Δ *boi2*Δ cells. This result suggests that Boi1 and Boi2 are required for Msb1 function.

Mild overproduction of Msb1 by high-copy *MSB1* did not cause bud elongation (Fig. 3B). To evaluate the functional relationship between Msb1 and Boi1/Boi2, we asked if an excess of Boi2 could enhance Msb1 function and thus lower the dosage of Msb1 required to cause bud elongation. To this end, we mildly overexpressed Boi2 under the control of *MET25* promoter in the cells. We found that mild overespression of Boi2 alone did not cause bud elongation except that 34% of cells (n = 200) became enlarged. In contrast, mild overexpression of Boi2 and Msb1 together caused bud elongation in about 10% of budded cells (n = 400), while the cell enlargement phenotype of Boi2-overproducing cells was not affected by high-copy *MSB1* (Fig. 3B). This result showed that Boi2 overproduction could lower the amount of Msb1 required to cause bud elongation, suggesting that Boi2 may normally function together with Msb1. Interestingly, mild overproduction of Bem1, which normally binds to Boi2 and functions as a scaffold promoting the assembly of Cdc24, Cdc42, and other proteins into a functional complex, together with Msb1 also produced elongated buds in budded cells albeit at a lower percentage (5%, n = 400) while mild overproduction of Cdc42 together with Msb1 failed to produce any elongated buds (Fig. S1). Unlike Boi2, Bem1 overproduction alone also produced elongated buds at a low percentage (2%, n = 400). Together, our findings indicate that the physical interaction observed between Msb1 and Boi1/Boi2 is functionally relevant. Msb1 may function together with Boi1/Boi2 and Bem1 in a protein complex to promote Cdc42 function.

### Overexpression of Msb1 Causes Defects in Cell Morphology, Septin Organization, and Cell Wall Synthesis

**Figure 4:**
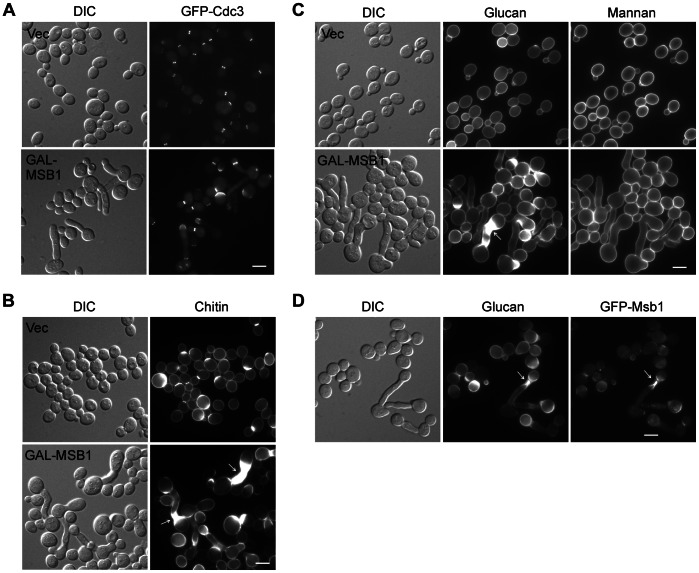
Phenotypes of cells overexpressing *MSB1*. (**A**) Morphology and septin organization (shown by GFP-Cdc3) in cells overexpressing *MSB1*. Cells of yeast strain JGY881 (*GFP-CDC3*) carrying pEGKT316 (Vec) or pEGKT316-MSB1 (GAL-MSB1) were grown in SC-Ura medium containing galactose and raffinose at 30°C for 16 h. DIC and GFP fluorescence images were taken. (**B**, **C**) Chitin deposition (**B**) as well as 1,3-β-glucan and mannan distribution (**C**) were visualized in cells of strain YEF473A carrying pEGKT316 (Vec) or pEGKT316-MSB1 (GAL-MSB1) grown in SC-Ura medium containing galactose and raffinose at 30°C. The cells were stained for chitin, 1,3-β-glucan, and mannan. (**D**) Msb1 localizes to sites of aberrant glucan deposition. Cells of strain JGY139A (*GAL1-GFP-MSB1*) was grown in SC-Ura medium containing galactose and raffinose at 30°C. Cells were stained for 1,3-β-glucan with aniline blue. Bars, 5 µm.

The lack of obvious phenotypes in *msb1*Δ cells imposes a major challenge for elucidating the physiological functions of Msb1. To gain more insight into the cellular functions of Msb1, we examined the effect of Msb1 overexpression on the growth and cell morphology in yeast cells. A global analysis of protein expression level in yeast cells identified Msb1 as a protein of low abundance [20]. According to this report, there are just about 172 molecules per cell for Msb1, which is significantly lower than the number of Bem1 (6490 molecules/cell), Cdc24 (1010 molecules/cell), Boi1 (1040 molecules/cell), and Boi2 (688 molecules/cell). Mild overexpression of *MSB1* under the control of its endogenous promoter on a high-copy plasmid did not impair cell growth or cell morphology (Fig. 3B), *MSB1* was then overexpressed under the control of a galactose-inducible promoter on a single-copy plasmid. A GST tag was fused to the N-terminus of Msb1. We found that overexpression of Msb1 did not cause defect in cell growth. However, 31% of budding cells (n>200) displayed morphological defects including elongated bud and multiple buds (usually two buds on one mother cell), which were not present in cells carrying empty vector or when the expression was repressed (on glucose-containing medium) (Fig. 4A, and data not shown). Among them, 12% of budding cells displayed one elongated bud; 6% of budding cells had multiple buds with normal shape; and 13% of budding cells exhibited both elongated buds and multiple buds.

The elongated-bud and multibudded morphology have been observed in mutants defective in septin organization, such as *elm1*Δ and *gin4*Δ mutants [23], [24]. To investigate if Msb1 overexpression might cause a defect in septin organization, we examined septin localization (indicated by GFP-Cdc3). We found that in cells that have a normal morphology, the septins were largely well organized at the mother-bud neck. However, in the cells that displayed morphological defects, septin organization was defective (Fig. 4A). The septins often showed reduced fluorescence or failed to organize into a tight hourglass structure at the bud neck. In the cells with an elongated bud, the septins were often found mislocalized to a patch at the bud tip. Bud elongation caused by overexpression of *MSB1* depends on Swe1, a bud neck-localized protein kinase that delays G_2_/M transition during the cell cycle [24], as overexpression of *MSB1* in *swe1*Δ cells failed to produce elongated buds (data not shown). This result suggests that Msb1 overexpression-induced bud elongation is a result of prolonged polarized growth due to G_2_/M delay.

Chitin deposition in cells overexpressing *MSB1* was also abnormal. Chitin is a component of yeast cell wall and is mainly deposited at the bud neck. By using calcofluor white, a fluorescent dye that binds chitin, we found that in cells overexpressing *MSB1*, chitin deposition at the bud neck was markedly increased in a large fraction of cells, mainly large-budded cells (Fig. 4B). Chitin was also deposited to a large region surrounding the bud neck in cells with elongated buds (Fig. 4B, arrows).

A previous study suggests that Msb1 is implicated in 1,3-β-glucan synthesis in the cell wall [19]. We then examined 1,3-β-glucan deposition by staining the cells with aniline blue, a fluorescent dye that specifically binds 1,3-β-glucan. We found that the distribution of 1,3-β-glucan was abnormal in Msb1-overexpressing cells (Fig. 4C, middle panel). In contrast, the distribution of α-mannan, another cell wall component, appears to be normal as shown by staining the cells with FITC-ConA, the fluorescein-conjugated lectin ConA (Fig. 4C, right panel). 1,3-β-Glucan was often found heavily deposited at the bud neck region, mainly in large-budded cells, but not in small-budded cells. In some of the elongated buds, increased glucan deposition occurred in a larger region surrounding the bud neck (Fig. 4C, middle panel, arrow). It is interesting to note that Msb1 and these aberrant glucan depositions co-localize as indicated by cells overexpressing GFP-Msb1 (Fig. 4D). In a number of elongated buds that subsequently formed a normal shaped bud at the tip, Msb1 was often found at the region surrounding the new bud neck (Fig. 4D, right panel, arrow), and heavy 1,3-β-glucan synthesis also occurred there. Surprisingly, we found that, at these locations, the cell wall appears to grow inward. In some cells, the cell wall grew so thick locally that it started to seal the narrow space near the bud neck (Fig. 4D, middle panel, arrow). Together, these results indicate that overexpression of Msb1 causes defects in cell morphology and septin organization as well as increased, aberrant chitin and glucan synthesis. More importantly, Msb1 appears to specifically stimulate cell wall synthesis at the bud neck in large-budded cells, i.e., during late stage of the cell cycle.

### Msb1 Inhibits Rho1 Function and Interacts with Rho1 *in vivo*

**Figure 5:**
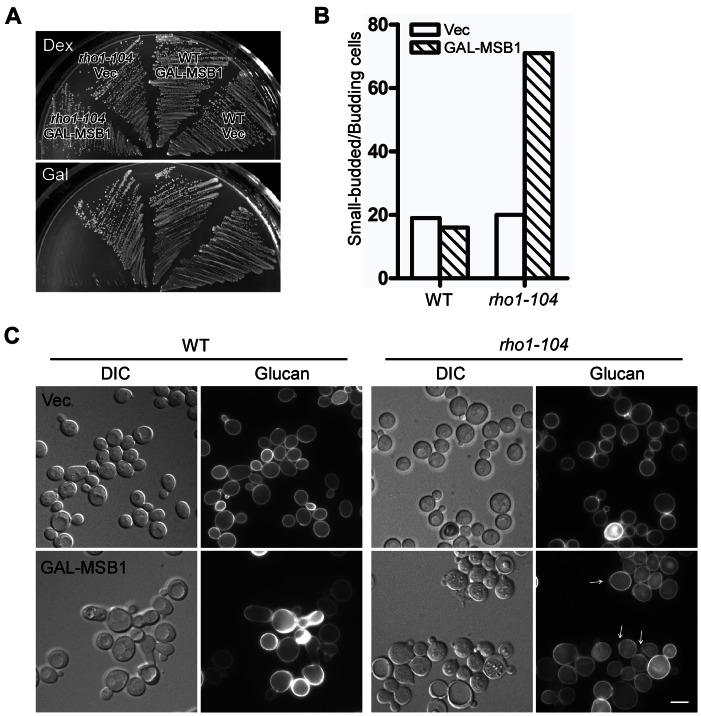
Msb1 overproduction inhibits the growth and glucan synthesis in *rho1-104* cells. (**A**) Overexpression of *MSB1* inhibits the growth of *rho1-104* cells. Cells of yeast strain NY1537 (WT) and NY1538 (*rho1-104*) carrying pEGKT316 (Vec) or pEGKT316-MSB1 (GAL-MSB1) were grown in SC-Ura medium containing dextrose (Dex) or galactose and raffinose (Gal) at 30°C. Pictures were taken after 3 d. (**B**) Overexpression of *MSB1* in *rho1-104* cells causes the accumulation of small-budded cells. The percentage of small buds in the population of budding cells overexpressing *MSB1* as in panel A was quantitated. More than 200 buds were scored. (**C**) Glucan distribution in *rho1-104* cells overexpressing *MSB1*. Cells as in panel A were stained for 1,3-β-glucan. Bar, 5 µm.

The aberrant glucan deposition in *MSB1*-overexpressing cells prompted us to examine if *MSB1* may genetically interact with *RHO1*. Interestingly, we observed that *GAL*-driven overexpression of *MSB1* severely impaired the growth of temperature-sensitive *rho1-104* cells, which are known to be primarily defective in cell wall synthesis [16], [17], at 30°C, a semi-permissive temperature (Fig. 5A). Microscopic examination revealed that *MSB1* overexpression in *rho1-104* cells caused dramatic accumulation of small-budded cells (Fig. 5B, 5C). When these cells were stained with aniline blue to reveal 1,3-β-glucan deposition in the cell wall, we found that aberrant glucan deposition was greatly reduced in *rho1-104* cells overexpressing *MSB1*. In small-budded cells, the bud cortexes of small buds were generally darker than those of the controls or medium- and large-budded cells (Fig. 5C, arrows), indicative of insufficient glucan synthesis. In contrast, glucan deposition in the bud cortexes of large-budded cells appears to be normal.

**Figure 6:**
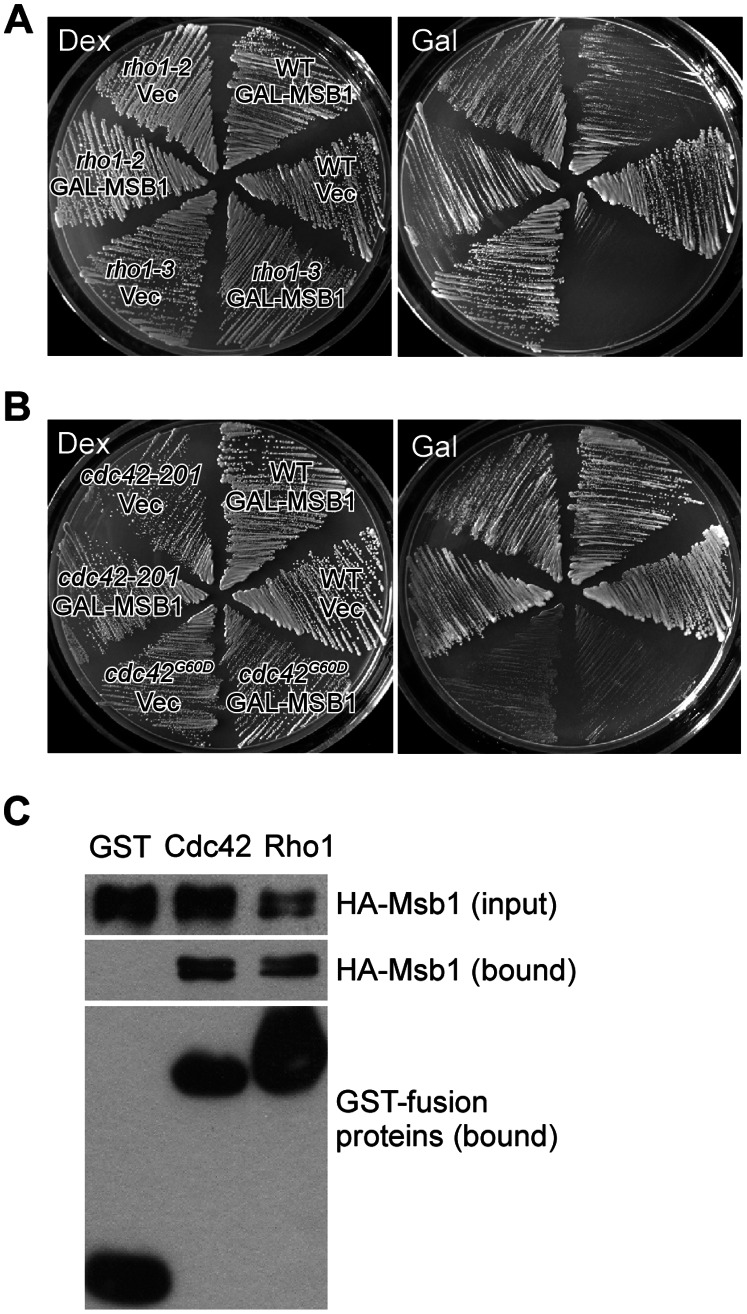
Msb1 inhibits Rho1 function and interacts with Rho1 *in vivo*. (**A**) Overexpression of *MSB1* inhibits the growth of *rho1-3* cells. Cells of yeast strain NY2284 (WT), NY2285 (*rho1-2*), and NY2286 (*rho1-3*) carrying pEGKT316 (Vec) or pEGKT316-MSB1 (GAL-MSB1) were grown in SC-Ura medium containing dextrose (Dex) or galactose and raffinose (Gal) at 30°C. Pictures were taken after 3 d. (**B**) Overexpression of *MSB1* does not inhibit the growth of *cdc42*-Ts cells. Cells of yeast strain YEF473A (WT), YEF2258 (*cdc42-201*), and JPC241 (*cdc42^G60D^*) carrying pEGKT316 (Vec) or pEGKT316-MSB1 (GAL-MSB1) were grown in SC-Ura medium containing dextrose (Dex) or galactose and raffinose (Gal) at 30°C. Pictures were taken after 4 d. (**C**) Msb1 interacts with Rho1 by GST pull-down assay. Cells of yeast strain YEF1395 (*msb1*Δ) carrying YEp181-3HA-MSB1 along with pEGKT (GST), pEGKT-CDC42 (Cdc42), or pEGKT-RHO1 (Rho1) were used in the assay. Molecular weight: GST (27 kDa), GST-Cdc42 (46 kDa), GST-Rho1 (48 kDa).

We also examined the effect of *MSB1* overexpression in two other *rho1*-Ts mutants, *rho1-2* and *rho1-3*. We observed that *MSB1* overexpression also markedly impaired the growth of *rho1-3* cells at 30°C, but not that of *rho1-2* cells (Fig. 6A). Compared to the growth inhibition by *MSB1* overexpression in *rho1-104* cells, the inhibition in *rho1-3* cells was less strong. Examination of 1,3-β-glucan synthesis in the cell wall revealed that glucan synthesis in the small buds of *rho1-3* cells overexpressing *MSB1* was reduced compared to that in wild-type cells (Fig. S2, arrows). Although aberrant deposition of 1,3-β-glucan persisted at the bud neck in *rho1-3* cells overexpressing *MSB1*, it occurred mainly in large-budded cells.

*MSB1* overexpression did not impair the growth of *cdc42-201* and *cdc42^G60D^* cells (Fig. 6B), suggesting that overproduction of Msb1 may specifically down-regulate Rho1 function. GST pull-down assay revealed that Msb1 interacts with Rho1 in yeast cells (Fig. 6C), suggesting that Msb1 may regulate Rho1 function by physically associating with Rho1.

Rho1 is known to control actin organization via Pkc1 and control cell wall 1,3-β-glucan synthesis via the glucan synthase, Fks1/Fks2. It was found that the *rho1-104* and *rho1-3* mutants are severely defective in the glucan synthase activity whereas the *rho1-2* mutant is primarily defective in the Pkc1-controlled pathway [15], [17]. The allele-specific inhibition by *MSB1* overexpression could be interpreted as that an excess of Msb1 may specifically affect the signaling of Rho1 to Fks1/Fks2, but not the signaling to Pkc1. This view is supported by the observation that the growth defect of *rho1-104* and *rho1-3* cells caused by Msb1 overproduction was suppressed by the addition of 1 M sorbitol into the medium (Fig. S3). Sorbitol was used here to provide osmotic support preventing cell lysis caused by insufficient cell wall synthesis. Therefore, our results suggest that Msb1 may down-regulate Rho1 function in cell wall synthesis. More importantly, the inhibition on Rho1 appears to occur specifically in the small bud at the small bud stage, i.e., during early stage of the cell cycle.

## Discussion

### Role of Msb1 in the Regulation of Cdc42 Function

Msb1 positively regulates Cdc42 function. However, Msb1 does not share sequence homology with either Cdc24 or Bem1. High-copy *MSB1* could not replace *CDC24* [8]. It also failed to suppress the growth defects of *bem1* and *bem2* mutants at 37°C [13]. Thus, it is not clear how Msb1 regulates Cdc42 function. For many years, no mechanistic insights into this important polarity protein are known. In this study, we report that Msb1 interacts with Cdc42 in yeast cells by GST pull-down assay (Fig. 1B). In addition, Msb1 interacts with Boi1 and Boi2 (Fig. 2A), two scaffold proteins that interact with Cdc42 and Bem1 and play an important role in the establishment of cell polarity [21], [22]. The interaction between Msb1 and Boi1/Boi2 is functionally relevant since an excess of Boi2 could enhance Msb1 function (Fig. 3B). Moreover, like *msb1*Δ, *boi1*Δ *boi2*Δ also displays synthetic lethality with *bem1* mutation [22]. These findings support the idea that Msb1 may function in the same pathway with Boi1 and Boi2. Interestingly, Msb1 interacts with the C-terminal portion of Boi1 and Boi2 that contains the Cdc42-interacting PH domain but lacks the Bem1-interacting proline-rich motif (Fig. 2B). This region of Boi2 is known to be essential for Boi2 function [21].

The interaction between Msb1 and Cdc42 is unlikely mediated by Boi1 and Boi2 because the interaction still occurs in *boi1*Δ *boi2*Δ cells (Fig. 2C). Msb1 may form a protein complex with Cdc42, Boi1, and Boi2 to promote the assembly of Cdc42, Cdc24, and other proteins into a functional complex. An increase of Msb1 production by high-copy *MSB1* in *cdc42* or *cdc24* mutants may cause the efficient assembly or functional signaling of this protein complex, leading to the rescue of the growth defect.

Boi1 and Boi2 are known to interact with Bem1 via the proline-rich motif of Boi1/Boi2 and the second SH3 domain of Bem1 [21], [22]. Although we failed to detect an association of Msb1 with Bem1 and Cdc24 by GST pull-down assay (Fig. 2A), we can not rule out the possibility that the Msb1-Boi1/Boi2 complex may function together with Bem1 to activate Cdc42 signaling. Msb1-Boi1/Boi2 could be in a larger protein complex that contains Bem1. Msb1-Boi1/Boi2 may support Bem1’s function. Conversely, Bem1 may also enhance the association of Cdc42 with Msb1 and Boi1/Boi2. In the absence of Bem1, the Msb1-Boi1/Boi2 complex could still operate but may be less efficient.

### Role of Msb1 in the Regulation of Rho1 Function

A previous study showed that *MSB1* on high-copy plasmid suppressed the growth defect of *rho1-2* cells [19], suggesting that Msb1 may positively regulate Rho1 function. We repeated the experiment but could not reproduce the same result (data not shown). In this study, we show that *GAL*-driven overexpression of Msb1 impaired the growth of *rho1-104* and *rho1-3* cells, which are severely defectively in the synthesis of 1,3-β-glucan in the cell wall, but not that of *rho1-2* cells. These results suggest that Msb1 may inhibit rather than promote Rho1 function.

Msb1 is known to positively regulate Fks1/Fks2, the 1,3-β-glucan synthase [19]. Our finding that *MSB1* overexpression caused increased deposition of 1,3-β-glucan at the bud neck as well as the co-localization of Msb1 and aberrant glucan deposition support this idea (Fig. 4C, 4D). While Msb1 acts positively on glucan synthesis, why does overexpression of *MSB1* inhibit the growth of *rho1-104* and *rho1-3* cells? The answer to this question may lie in the small buds of small-budded cells. We find that Msb1 overproduction in *rho1-104* cells caused a dramatic increase of small-budded cells in the population of budding cells and reduced 1,3-β-glucan synthesis in the small buds, but not in large-budded cells. In contrast, *MSB1* overexpression stimulated glucan synthesis at the bud neck mainly in those large-budded cells. Consider that Msb1 localizes to the bud cortex in small-budded cells and to the bud neck in large-budded cells (Fig. 1A), Msb1 may specifically down-regulate Rho1 function at the bud cortex during early stage of the cell cycle when the bud is small but promote glucan synthesis at the bud neck during late stage of the cell cycle. Thus, the inhibition of Rho1 function in glucan synthesis and the stimulation of Fks1/Fks2 are temporally separated during bud development. The inhibition of Rho1 function could be mediated by a physical binding of Msb1 to Rho1. Our finding that Msb1 interacts with Rho1 in GST pull-down assay supports this idea.

### Role of Msb1 in the Coordination of Cdc42 and Rho1 Functions during Early Bud Development

Cdc42 and Rho1 both play essential roles during polarized growth in small-budded cells. Cdc42 is required for polarized actin organization as well as secretion in the emerging bud [2], [3], [25], whereas Rho1 is critical for cell wall synthesis [17], [18]. They are both present on the bud cortex in small-budded cells [6], [16] and share some common downstream effectors, such as Bni1 (formin) and Sec3 (a subunit of exocyst). In a previous study, we reported that overexpression of *CDC42* inhibited the growth of *rho1*-Ts mutants and *vice versa* [10], suggesting that Cdc42 and Rho1 could be functionally antagonistic to each other. Cdc42 and Rho1 may act cyclically to promote bud growth: Cdc42 directs polarized secretion, which may loosen up the bud tip and allow the expansion of bud surface. Rho1, on the other hand, may direct the local synthesis of 1,3-β-glucan in the newly expanded bud surface to protect the bud from lysis. Thus, a balance of Cdc42 and Rho1 activity may be required to ensure proper bud development.

How are Cdc42 and Rho1 functions properly coordinated during bud development? We previously suggested that Pxl1, a paxillin-like protein, may play a role in this process because overproduction of Pxl1 rescued the growth defect of *cdc42-201* and *cdc42^G60D^* mutants but inhibited the growth of *rho1-2* and *rho1-3* mutants [10]. Because the deletion of *PXL1* did not abolish the reciprocal inhibition between Cdc42 and Rho1, we suspect that other mediators might exist for this function. Msb1 is known to promote Cdc42 function. In this study, we show that Msb1 interacts with Cdc42 and Rho1 in the cells. In addition, Msb1 inhibits Rho1 function specifically in small-budded cells. Thus, Msb1 may play a role in the coordination of Cdc42 and Rho1 functions during early stage of bud development.

Msb1 carries a RhoGAP domain at its N-terminus. This region is highly homologous among Msb1 homologs in other yeast species and filamentous fungi. Although the observation that high-copy *MSB1* suppressed rather than impaired the growth of several *cdc42*-Ts mutants suggests that Msb1 may not function as a RhoGAP for Cdc42, we find that the RhoGAP domain is required for the suppression of *cdc42-201* mutant by high-copy *MSB1* (our unpublished data). We speculate that the RhoGAP domain may constitute a binding surface for the interaction of Msb1 with Cdc42 and Rho1. One good example is the mammalian IQGAPs, which bind to mammalian Cdc42 through the RasGAP domain, but itself does not display GAP activity towards Cdc42 [26]. Future studies will be needed to reveal the molecular basis for the interaction of Msb1 with Cdc42 and Rho1.

## Materials and Methods

### Strains and Genetic Methods

Yeast strains used in this study are listed in Table 1. Standard culture media and genetic techniques were used except where noted [27]. For the induction of *GAL*-driven overexpression of *MSB1*, yeast cells were grown in SC-Ura medium containing 2% of galactose and 1% of raffinose. *Escherichia coli* strains DH12S (Life Technologies, Gaithersburg, MD) were used as hosts for plasmid manipulation. Oligonucleotide primers for PCR were ordered from Sangon Biotech (Shanghai, China). The nucleotide sequences of primers are available upon request.

**Table 1:** *S. cerevisiae* strains used in this study.

Strain	Genotype	Source
YEF473A	*MAT*a *his3-*Δ*200 leu2-*Δ*1 lys2-801 trp1-*Δ*63 ura3-52*	[32]
YEF473B	*MAT*α *his3-*Δ*200 leu2-*Δ*1 lys2-801 trp1-*Δ*63 ura3-52*	[32]
YEF1395	*MAT*a *his3-*Δ*200 leu2-*Δ*1 lys2-801 trp1-*Δ*63 ura3-52 msb1*Δ*::HIS3*	This study
YEF2258	*MAT*a *his3-*Δ*200 leu2-*Δ*1 lys2-801 trp1-*Δ*63 ura3-52 cdc42-201*	[25]
JGY139A	*MAT*a *his3-*Δ*200 leu2-*Δ*1 lys2-801 trp1-*Δ*63 ura3-52 GAL1-GFP-MSB1:HIS3MX*	This study
JGY881	*MAT*a *his3-*Δ*200 leu2-*Δ*1 lys2-801 trp1-*Δ*63 ura3-52 GFP-CDC3:LEU2*	[30]
JGY2349	*MAT*a *his3-*Δ*200 leu2-*Δ*1 lys2-801 trp1-*Δ*63 ura3-52 boi2*Δ*::TRP1*	This study
JGY2425	*MAT*α *his3-*Δ*200 leu2-*Δ*1 lys2-801 trp1-*Δ*63 ura3-52 boi1*Δ*::HIS3MX*	This study
JGY2821	*MAT*a *his3-*Δ*200 leu2-*Δ*1 lys2-801 trp1-*Δ*63 ura3-52 boi1*Δ*::HIS3MX boi2*Δ*::TRP1*	This study
JPC241	*MAT*a *his3-*Δ*200 leu2-*Δ*1 lys2-801 trp1-*Δ*63 ura3-52 cdc42^G60D^*	[33]
NY1537	*MAT*a *ade2 his3 leu2 trp1 ura3*	[34]
NY1538	*MAT*a *ade2 his3 leu2 trp1 ura3 rho1-104*	[34]
NY2284	*MAT*α *ade2 his3 leu2 lys2 trp1 ura3 rho1::HIS3 ade3::*[pRHO1-*RHO1:LEU2*]	[34]
NY2285	*MAT*a *ade2 his3 leu2 lys2 trp1 ura3 rho1::HIS3 ade3::*[pRHO1-*rho1-2:LEU2*]	[34]
NY2286	*MAT*α *ade2 his3 leu2 lys2 trp1 ura3 rho1::HIS3 ade3::*[pRHO1-*rho1-3:LEU2*]	[34]

### Plasmid Construction

Plasmid YEp181-3HA-MSB1 was constructed by several steps. First, the 5.31 kb *Bam*HI fragment from the genomic insert of YEp13-MSB1 [8] was inserted into YIplac211. The *MSB1* gene in this plasmid contains a 1203 bp promoter. Then, a *Not*I site was introduced into *MSB1* right after the ATG start codon by site-directed DNA mutagenesis, yielding YIp211-MSB1-NotI. A *Not*I fragment encoding the 3HA epitope [28] was then inserted into the *Not*I site of YIp211-MSB1-NotI, yielding YIp211-3HA-MSB1. Lastly, the 5.31 kb *Hin*dIII-*Sac*I 3HA-MSB1 fragment was inserted into YEplac181, yielding YEp181-3HA-MSB1 (2μ *LEU2*). YEp181-GFP-MSB1 was generated by replacing the 3HA epitope in YEp181-3HA-MSB1 with a *Not*I fragment encoding GFP (S65T). pRS426-GFP-MSB1 was generated by inserting the *Sac*I-*Sal*I fragment containing GFP-MSB1 from YEp181-GFP-MSB1 into pRS426 (2μ *URA3*).

Plasmids pEGKT-CDC42, pEGKT-CDC42^Q61L^, pEGKT-CDC42^T17N^, pEGKT-CDC24, pEGKT-BEM1, and pEGKT-RHO1 for GST pull-down assay were described previously [29]. To generate pEGKT-BOI1, pEGKT-BOI2, pEGKT-BOI1-C, and pEGKT-BOI2-C, full-length *BOI1* and *BOI2* or *BOI1-C* and *BOI2-C* were amplified by PCR from the genomic DNA of yeast strain YEF473A, digested by *Bam*HI and *Sal*I, and ligated into pEGKT (2µ, *URA3*, *UAS_GAL1_-P_CYC1_-GST*). Plasmid pEGKT316-MSB1 was constructed by inserting the *Bam*HI-*Hin*dIII fragment of *MSB1* ORF amplified from YEp13-MSB1 by PCR into pEGKT316 (*CEN*, *URA3,UAS_GAL1_-P_CYC1_-GST-T_CYC1_*) [30]. pUG36-BOI2 was constructed by inserting full-length *BOI2* ORF amplified by PCR from genomic DNA into *Bam*HI- and *Sal*I- digested pUG36 (*CEN*, *URA3*, *P_MET25_-yEGFP3-T_CYC1_*) [10].

### Yeast Strain Construction

The yeast strains JGY1395 (a *msb1*Δ::*HIS3MX*), JGY2349 (a *boi2*Δ::*TRP1*), and JGY2425 (α *boi1*Δ::*HIS3MX*) were constructed by the deletion of *MSB1*, *BOI2*, or *BOI1* gene in yeast strain YEF473A or YEF473B by a PCR-based method [31]. For the construction of JGY139A (a *GAL1-GFP-MSB1:HIS3MX*), the *HIS3MX-P_GAL1_-GFP(S65T)* module was inserted into the chromosomal *MSB1* locus of yeast strain YEF473A just before the ATG start codon by a PCR-based method [31]. For the construction of JGY2821, yeast strains JGY2349 (a *boi2*Δ::*TRP1*) and JGY2425 (α *boi1*Δ::*HIS3MX*) were crossed to form a diploid. The diploid strain was then sporulated and tetrads were dissected to yield the *boi1*Δ *boi2*Δ strain. It takes more than 10 days for the *boi1*Δ *boi2*Δ segregants to germinate and grow into a colony on the YPD dissection plate at 22°C.

### GST Pull-down Assay

The assay follows a protocol as previously described [29], [30]. Standard immunoblotting procedure was used. HA-Msb1 and GST-fusion proteins were resolved in 7.5% and 12.5% SDS-PAGE gel, respectively. Primary antibodies used were mouse monoclonal antibodies against HA and GST (Covance Research Products, Richmond, CA). Horseradish peroxidase-conjugated goat anti-mouse IgG was used as secondary antibody.

### Microscopy

An Olympus BX51 microscope (Tokyo, Japan) and a Retiga 2000R CCD camera (QImaging Corporation, Canada) were used in the visualization of cell morphology and GFP-tagged proteins by differential interference contrast (DIC) and fluorescence microscopy. The images were obtained using QCapture Suite (QImaging Corporation, Canada). ImagePro Plus (Glen Mills, PA) was used for image processing. To stain yeast cells for 1,3-β-glucan, chitin, or mannan in the cell wall, yeast cells were incubated with 0.05% aniline blue (Acros Organics, New Jersey), 0.01% calcofluor white (Sigma-Aldrich), or 100 µg/ml FITC-ConA (Molecular Probes, Oregon), respectively, for 5 min at 24°C.

## Supporting Information

Figure S1 Morphology of cells with elevated expression of *BEM1* and *MSB1* as well as *CDC42* and *MSB1*. (**A**) Cells of yeast strain YEF473A carrying plasmids YEp13-MSB1/YEp24 (MSB1↑), YEp13/YEp24-BEM1 (BEM1↑), or YEp13-MSB1/YEp24-BEM1 (MSB1↑ BEM1↑) were grown on SC-Leu-Ura plate containing dextrose at 30°C for 16 h. (**B**) Cells of yeast strain YEF473A carrying plasmids YEp13-MSB1/YEp24 (MSB1↑), YEp13/YEp24-CDC42 (CDC42↑), or YEp13-MSB1/YEp24-CDC42 (MSB1↑ CDC42↑) were grown on SC-Leu-Ura plate containing dextrose at 30°C for 16 h. Bars, 5 µm.
(TIF)

Figure S2 Glucan distribution in NY2284 (WT), NY2285 (*rho1-2*), and NY2286 (*rho1-3*) cells overexpressing *MSB1*. Cells carrying pEGKT316 (Vec) or pEGKT316-MSB1 (GAL-MSB1) were grown in SC-Ura medium containing galactose and raffinose at 30°C for 3 d and stained for 1,3-β-glucan. Bar, 5 µm.
(TIF)

Figure S3 The growth defect of *rho1-104* and *rho1-3* cells caused by Msb1 overproduction can be suppressed by 1 M sorbitol. (**A**) Cells of yeast strain NY1537 (WT) and NY1538 (*rho1-104*) carrying pEGKT316 (Vec) or pEGKT316-MSB1 (GAL-MSB1) were grown in SC-Ura medium containing galactose (Gal) or galactose plus 1 M sorbitol (Gal+Sorbitol) at 30°C. Pictures were taken after 3 d. (B) Similar to (A), cells of strain NY2284 (WT) and NY2286 (*rho1-3*) carrying pEGKT316 (Vec) or pEGKT316-MSB1 (GAL-MSB1) were grown at 30°C. Pictures were taken after 4 d.
(TIF)
