# Supplementary figures and images for: Msb1 Interacts with Cdc42, Boi1, and Boi2 and May Coordinate Cdc42 and Rho1 Functions during Early Stage of Bud Development in Budding Yeast
Source: PLoS One. 2013 Jun 13;8(6):e66321. doi: 10.1371/journal.pone.0066321 (PMC3681933; doi:10.1371/journal.pone.0066321)

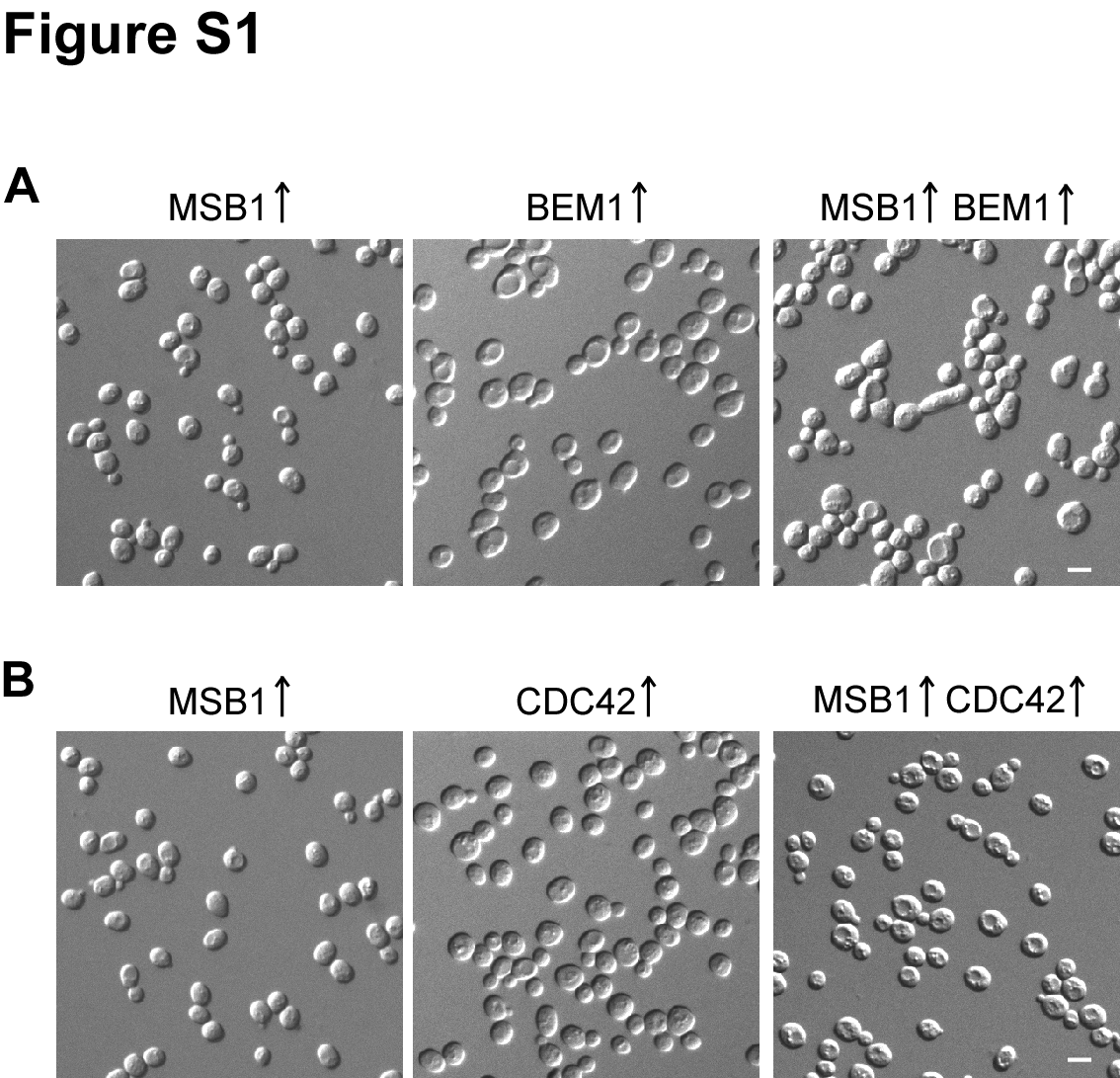

Supplement: Figure S1 — Morphology of cells with elevated expression of BEM1 and MSB1 as well as CDC42 and MSB1. (A) Cells of yeast strain YEF473A carrying plasmids YEp13-MSB1/YEp24 (MSB1↑), YEp13/YEp24-BEM1 (BEM1↑), or YEp13-MSB1/YEp24-BEM1 (MSB1↑ BEM1↑) were grown on SC-Leu-Ura plate containing dextrose at 30°C for 16 h. (B) Cells of yeast strain YEF473A carrying plasmids YEp13-MSB1/YEp24 (MSB1↑), YEp13/YEp24-CDC42 (CDC42↑), or YEp13-MSB1/YEp24-CDC42 (MSB1↑ CDC42↑) were grown on SC-Leu-Ura plate containing dextrose at 30°C for 16 h. Bars, 5 µm. (TIF) [file pone.0066321.s001.tif]

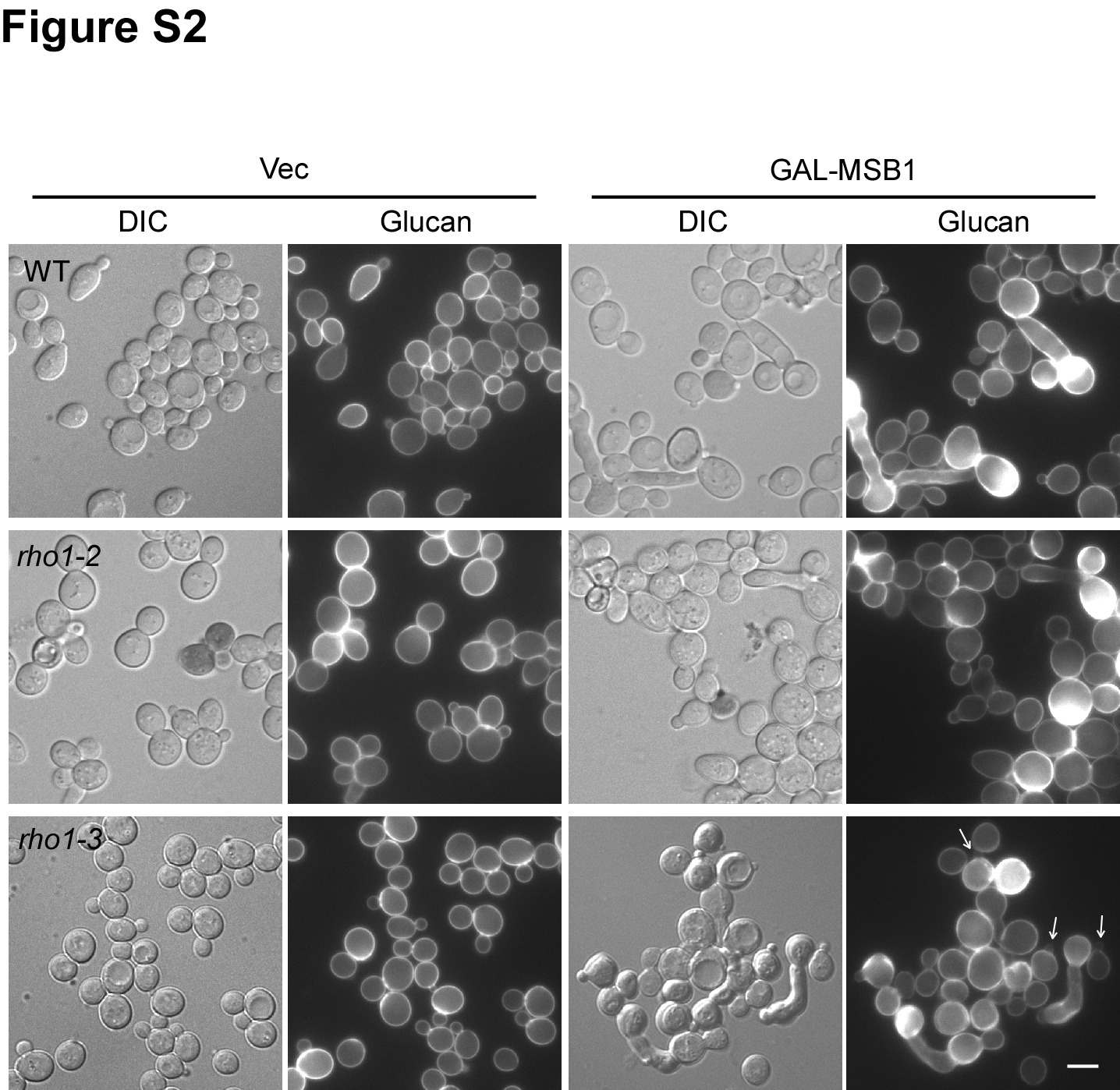

Supplement: Figure S2 — Glucan distribution in NY2284 (WT), NY2285 (rho1-2), and NY2286 (rho1-3) cells overexpressing MSB1. Cells carrying pEGKT316 (Vec) or pEGKT316-MSB1 (GAL-MSB1) were grown in SC-Ura medium containing galactose and raffinose at 30°C for 3 d and stained for 1,3-β-glucan. Bar, 5 µm. (TIF) [file pone.0066321.s002.tif]

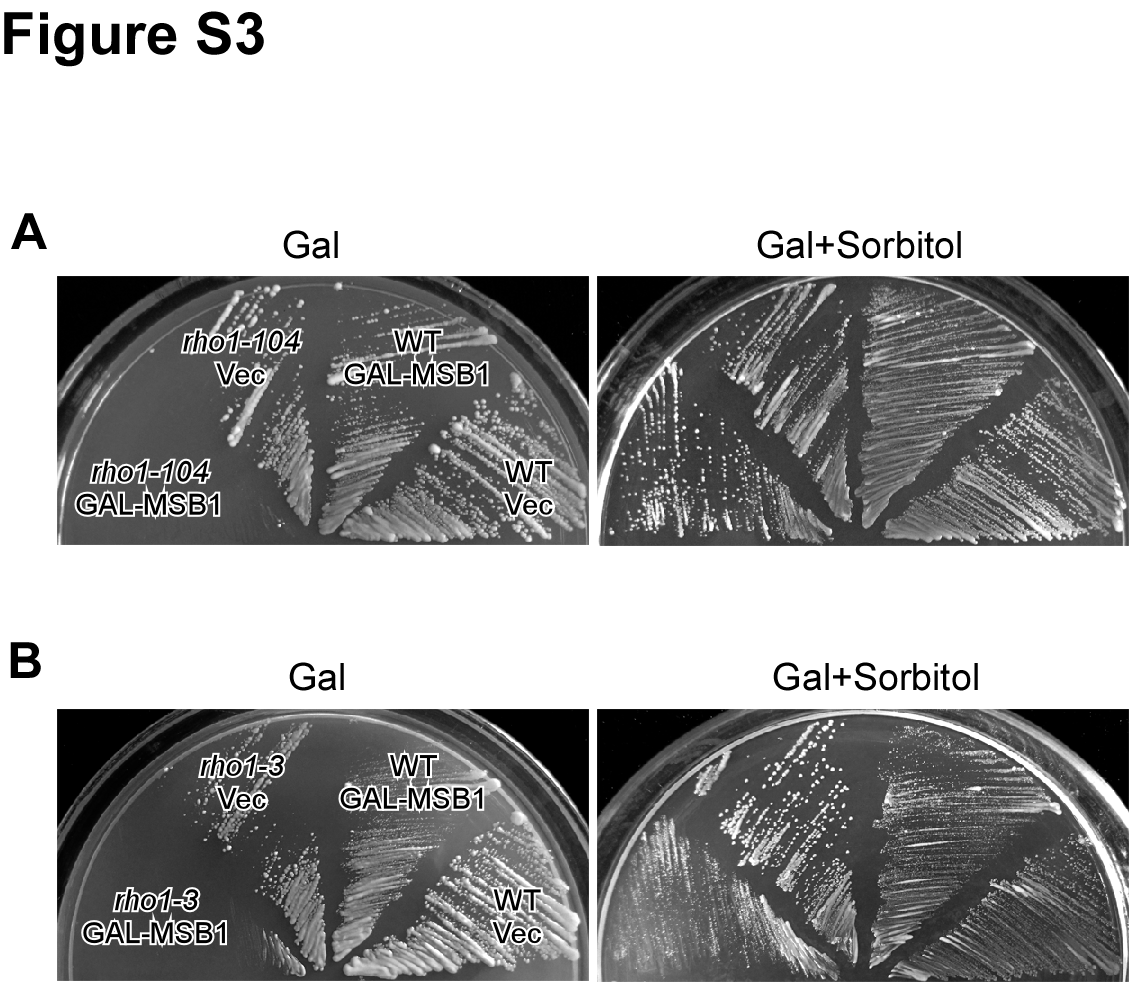

Supplement: Figure S3 — The growth defect of rho1-104 and rho1-3 cells caused by Msb1 overproduction can be suppressed by 1 M sorbitol. (A) Cells of yeast strain NY1537 (WT) and NY1538 (rho1-104) carrying pEGKT316 (Vec) or pEGKT316-MSB1 (GAL-MSB1) were grown in SC-Ura medium containing galactose (Gal) or galactose plus 1 M sorbitol (Gal+Sorbitol) at 30°C. Pictures were taken after 3 d. (B) Similar to (A), cells of strain NY2284 (WT) and NY2286 (rho1-3) carrying pEGKT316 (Vec) or pEGKT316-MSB1 (GAL-MSB1) were grown at 30°C. Pictures were taken after 4 d. (TIF) [file pone.0066321.s003.tif]
